# Supplementary material for: Initial Study on the Impact of Probiotics on Postoperative Gastrointestinal Symptoms and Gut Microbiota after Sleeve Gastrectomy: A Placebo-Controlled Study
Source: Nutrients. 2024 Oct 15;16(20):3498. doi: 10.3390/nu16203498 (PMC11510060; doi:10.3390/nu16203498)
Supplement: Supplementary file 1 [file nutrients-16-03498-s001.zip › nutrients-3239981-supplementary.pdf]

	Candida krusei		*Candida albicans*		*Candida tropicalis*		*Candida glabrata*		*Candida* spp.		Molds		*Escherichia coli*		*Carbapenemase-Producing Escherichia coli (CPE)*	
Patients	pre	post	pre	post	pre	post	pre	post	pre	post	pre	post	pre	post	pre	post
P1	100	100	100	100	100	100	100	100	100	100	100	100	1240000	2400000	100	100
P2	100	100	100	100	100	100	100	100	100	2000	100	100	2000000	110000000	100	100
P3	100	100	26000	100	100	100	2000	100	28000	6000	100	100	3300000	30000000	100	100
P4	100	100	100	26000	100	100	100	100	100	52000	100	100	34000000	14000000	100	100
P5	100	100	100	100	100	100	100	100	100	800	100	100	84000000	94000000	100	100
P6	100	2000	100	100	100	100	100	16000	100	16000	100	100	2000000	16000000	100	1000
P7	100	100	6000	28000	100	100	100	100	6000	28000	100	100	8000000	6000000	100	1000
P8	100	100	400	100	100	100	400	100	1400	2000	100	100	2000000	8000000	100	1000
P9	100	100	100	100	100	100	100	100	100	100	100	100	14000000	42000000	100	1000
P10	100	100	100	2000	100	100	100	100	400	2000	100	100	1000	26800	100	1000
P11	100	100	2400	100	100	100	100	100	4000	4000	100	100	14000000	1000	100	1000
P12	100	100	32000	200	100	100	100	100	50000	600	100	100	332000000	580000000	100	1000
P13	100	100	400	3800	100	100	100	100	1400	33200	100	100	10000000	1200000	1000	1000
P14	100	100	100	100	100	100	100	100	100	100	100	100	2800000	400000000	1000	1000
C1	100	100	200	100	100	100	100	100	200	100	100	100	520000000	184000000	24600000	100
C2	100	100	100	100	100	100	100	100	100	100	100	100	2140000	940000000	100	100
C3	100	100	20000	400	100	100	100	100	38000	1000	100	100	8000000	6000000	100	100
C4	100	100	128000	100	100	100	100	100	226000	700	100	100	24000000	36000000	100	100
C5	100	100	4000	400	100	100	100	100	4000	400	100	100	5000000	40000000	100	1000
C6	100	100	2000	4000	2000	100	100	100	6200	4000	100	4000	2000000	620000000	100	1000
C7	100	100	100	100	100	100	100	100	2000	100	100	100	38000	60000000	100	1000
C8	100	100	400	6000	100	100	100	100	400	6000	100	100	28000000	60000000	100	1000
C9	100	100	400	100	100	100	100	100	400	100	100	100	8800000	60000000	100	1000
C10	100	100	4000	100	100	100	100	100	6000	4000	100	100	58000000	80000000	100	1000
C11	100	100	100	100	100	100	100	100	100	200	100	100	52000	42000000	100	1000
C12	100	100	100	100	100	100	100	100	100	100	100	100	600000	16000000	100	1000
C13	100	100	100	100	100	100	100	100	100	100	100	100	2400000	400000000	1000	1000
C14	100	100	100	6000	100	100	100	100	100	6000	100	100	80000000	1000	1000	1000
p	n/a	0.327	0.575	0.255	0.362	n/a	0.223	0.327	0.482	0.052	n/a	0.327	0.812	0.325	0.362	0.699

No	*Pseudomonas aeruginosa*		*Klebsiella pneumoniae*		*Carbapenemase-producing Klebsiella pneumoniae (CPE)*		*Enterococcus spp.* resistant to vancomycin		*Enterococcus* spp.		*Enterococcus faecalis*		*Enterococcus faecium*	
	pre	*post*	*pre*	*post*	*pre*	*post*	*pre*	*post*	*pre*	*post*	*pre*	*post*	*Pre*	*post*
P1	100	100	400000	40000	400000	100	200000	100	200000	54000000	1000	1000	4000	10000000
P2	100	100	20000	1000	2000	100	100	100	120000	8000000	1000	1000	48000	2000000
P3	100	100	100	8000000	100	100	100	100	2500000	14000000	1000	1000	1400000	4000000
P4	100	100	1000	4000	100	100	100	100	1000	20000	1000	2000	1000	16000
P5	100	100	1000	1000	100	100	100	100	4200000	46000	1000	1000	4000000	2000
P6	100	100	54000	100000000	100	1000	100	100	4600000	3600000	1000	200000	2800000	1000
P7	100	100	4000000	4000000	100	1000	100	100	24000000	160000000	1000	1000	384000	46000000
P8	100	100	1000	1000	100	1000	100	100	8000000	80000000	2000	80000000	6000000	1000
P9	100	100	1000	600000	100	1000	100	100	10000000	16000000	80000	1000	120000	14000000
P10	100	100	1000	1000	100	1000	100	1000	200000	1360000	38000	48000	1000	1000
P11	100	100	1000	1000	100	1000	100	1000	1000	1400000	1000	400	2000	4000
P12	100	100	1000	1000	100	1000	100	1000	200000000	4000000	2000000	4000000	108000000	1000
P13	100	100	1000	200000	1000	1000	100	1000	20000000	200000000	2000	20000	4000	4000000
P14	100	100	1000	22000000	1000	1000	1000	1000	166000	80000000	10000	36000000	1000	8000000
C1	100	100	100	1000	100	100	100	100	480000	204000000	1000	20000000	400000	114000000
C2	100	100	1000	1000	100	100	100	6200	780000	62000000	1000	1000	352000	200000
C3	100	100	1000	1000	100	100	100	100	2000	200000	1000	1000	2000	1000
C4	100	100	1000	1000	100	100	100	100	140000	200000	1000	1000	44000	32000
C5	100	100	100	200000	100	1000	100	100	4000	10000000	1000	1000	1000	400000
C6	100	100	666000000	340000000	100	1000	100	100	1600000	20000000	1000	46000000	92000	1000
C7	100	100	1000	12000000	100	1000	100	100	106000	7200000	1000	8000	1000	2000000
C8	100	100	1000	1000	100	1000	100	1000	2000000	120000000	16000	6000000	120000	72000000
C9	42000	9400000	6000	20000000	100	1000	100	1000	400000	8000	1000	1000	32000	2000
C10	100	100	1000	1000	100	1000	100	1000	400000	1000000	4000	318000	100	1000
C11	100	100	1000	1000	100	1000	100	1000	100000	600000	12000	110000	1000	16000
C12	100	100	22000	1800000	100	1000	100	1000	12000	110000000	1000	600000	12000	80000000
C13	100	100	1000	1000	1000	1000	100	1000	78000	200000	1000	1000	1000	6000
C14	100	100	200000	420000000	1000	1000	1000	1000	4000000	48000	1000	100	6000	48000
p	0.362	0.327	0.365	0.221	0.290	0.699	0.295	0.206	0.157	0.799	0.270	0.633	0.231	0.245

No	*Salmonella* spp.		*Proteus mirabilis*		*Staphylococcus aureus*		*Staphylococcus saprophyticus*		*Clostridium perfringens*		*Clostridium difficile*		Other *Clostricium spp*	
	pre	*post*	*pre*	*post*	*pre*	*post*	*pre*	*post*	*pre*	*post*	*pre*	*post*	*Pre*	*post*
P1	100	100	700	40000	100	2000	100	100	9400000	800000	100	100	3200	800000
P2	100	100	600	60000	100	100	100	2000	4000	9400000	100	100	200000	21400000
P3	100	100	72000	100000	100	100	100	100	16000	5600000	100	100	100	9600000
P4	0	100	0	800000	100	300	100	100	18000	332000	100	100	58000	200000
P5	0	100	0	10000000	100	100	100	100	32000000	400000	3000000	540000	100	14799900
P6	100	100	100	100	20000	234000	100	100	16000	100	100	100	200000	2400000
P7	100	100	312000	56000	1000	100	1000	100	24000	600000	100	16000	1200000	11000000
P8	100	100	100	200000	4000	200	100	100	100	100	100	100	600000	20000
P9	100	100	100	1800000	100	100	100	100	52000	2000	100	100	28200000	6200000
P10	100	100	100	100	100	2400	100	100	100	0	100	100	10000	1000000
P11	100	100	1000000	0	100	100	68000	100	100	200	100	100	5200000	1200000
P12	100	100	10000000	0	100	100	100	100	8000	134000	100	100	4000000	96000000
P13	100	100	6200000	0	100	100	100	2000	26000	34000	100	100	19000000	19600000
P14	100	100	438000	0	100	100	14000	100	100	74000	100	100	15600000	1400000
C1	100	0	46000	100000	100	12000	100	100	800000	8000000	<100	100	400000	16000000
C2	100	100	2000	1000000	100	400	100	200	100	1400000	100	100	1000000	2800000
C3	100	100	600	44000	100	100	100	100	300	8000	100	100	1000000	1000000
C4	0	100	0	8800000	100	100	100	100	14000	8800000	100	100	600000	400000
C5	100	100	138000	800000	1000	100	1000	100	100	6000	14800	100	5600000	14000000
C6	100	100	100	400000	1000	100	1000	100	100	16800000	170000	308000	4000000	100
C7	100	100	100	2600000	10000	2000	1000	100	18000	1900000	100	100	200000	100
C8	100	100	100	400000	100	32000	100	100	40000	200000	100	100	800000	600000
C9	100	100	44000	600000	100	100	100	100	1400000	6800000	100	100	1200000	13400000
C10	100	100	4000	1600000	100	100	100	100	200	24000	100	100	124000	14200000
C11	100	100	6000	0	100	100	2000	100	18000	1800000	100	100	2400000	3400000
C12	100	100	12000	0	100	100	100	100	4000	4000	100	100	2000000	400000
C13	100	100	800000	0	100	100	100	100	1000	100	100	100	130000	2000000
C14	100	100	4600000	0	100	100	100	100	100	100	100	100	400000	200
p	0.476	0.327	0.283	0.805	0.486	0.422	0.419	0.164	0.203	0.198	0.337	0.693	0.403	0.233

No	Total number of bacteria grown in anaerobic conditions		Incl. *Bifidobacterium* spp.		Total number of bacteria grown in aerobic conditions		pH	
	pre	*post*	*pre*	*post*	*pre*	*post*	*pre*	*post*
P1	300000000	800000000	10000	100000	1520000	40000000	6,70	7,32
P2	1000000000	200000000	10000	20000000	2000000	0	5,94	6,93
P3	500000	400000000	10000	200000000	20000000	58000000	6,80	6,89
P4	1100000000	220000000	400000000	60000000	34000000	8400000	6,36	5,85
P5	1200000000	100000000	400000000	20000000	84000000	160000000	7,23	6,91
P6	160000000	400000000	40000000	100000	22000000	120000000	6,54	7,10
P7	2700000000	300000000	1700000000	100000	128000000	240000000	5,54	6,90
P8	340000000	340000000	100000	100000	12000000	100000000	6,39	7,03
P9	1600000000	800000000	600000000	100000000	40000000	100000000	7,00	7,34
P10	60000000	50000000	40000000	100000	1000000	8000000	6,61	7,73
P11	200000000	120000000	100000	100000	34000000	1760000	6,63	7,23
P12	5400000000	6300000000	1000000000	100000	380000000	1000000000	7,32	7,50
P13	700000000	900000000	200000000	200000000	20000000	600000000	6,29	5,90
P14	2700000000	2100000000	2000000000	100000	12000000	600000000	7,74	6,87
C1	4000000000	240000000	10000	100000	2000000	900000000	6,31	6,73
C2	600000000	3000000000	10000	100000	2140000	1640000000	6,10	8,40
C3	140000000	140000000	10000	100000	8000000	12000000	6,57	6,06
C4	18000000000	1200000000	300000000	40000000	60000000	80000000	6,49	6,77
C5	2200000000	1500000000	200000000	700000000	16000000	20000000	6,78	6,49
C6	2100000000	1300000000	1000000000	100000	1690000000	900000000	6,39	7,64
C7	1900000000	800000000	1000000000	700000000	200000	140000000	6,53	5,97
C8	6200000000	1900000000	400000000	200000	40000000	20000000	6,82	6,38
C9	600000000	200000000	200000000	100000	8800000	120000000	7,64	7,25
C10	40000000	800000000	100000	100000000	60000000	96000000	5,85	7,52
C11	40000000	440000000	100000	100000000	640000	62000000	7,95	6,88
C12	600000000	1100000000	300000000	40000000	40000000	198000000	6,04	6,64
C13	400000000	600000000	20000000	100000000	20000000	460000000	6,25	7,15
C14	1500000000	800000000	200000000	100000	50000000	600000000	6,13	5,50
p	0.237	0.885	0.854	0.230	0.551	0.307	0.739	0.543
